# Evolution of Neuroparasitic Infections in the South of Madrid Region, Spain: A Hospital-Based Study

**DOI:** 10.3390/pathogens15101027

**Published:** 2026-09-30

**Authors:** Víctor Antón-Berenguer, Jorge Ligero-López, Alexandra Martín Ramírez, Antonio Nicolás Ruiz Benito, María Dolores Martín Rodrigo, Sara Gómez de Frutos, Sara María Quevedo Soriano, Francisco Jesús Merino Fernández, Maria Delmans Flores-Chavez, José Miguel Rubio Muñoz

**Affiliations:** 1 Microbiology and Parasitology Department, Severo Ochoa University Hospital, Av. de Orellana, s/n, 28914 Leganés, Spain; victor.antonb@gmail.com (V.A.-B.); antocolas.ruiz@gmail.com (A.N.R.B.); dmrodrigo@salud.madrid.org (M.D.M.R.); sara.gomezde@salud.madrid.org (S.G.d.F.); saramaria.quevedo@salud.madrid.org (S.M.Q.S.); fjmefer@gmail.com (F.J.M.F.); 2 Reference and Research Laboratory for Parasitology, National Centre for Microbiology, Instituto de Salud Carlos III (ISCIII), Ctra. Majadahonda Pozuelo Km.2, 28220 Madrid, Spain; a.martin@isciii.es; 3 Clinical Microbiology Department, Hospital Universitario Miguel Servet, Pº de Isabel la Católica, 1-3, 50009 Zaragoza, Spain; ligero999@hotmail.com; 4 CIBER de Enfermedades Infecciosas (CIBERINFEC), Instituto de Salud Carlos III, 28220 Madrid, Spain; 5 Mundo Sano Foundation, Calle Recaredo, 3, 28002 Madrid, Spain

**Keywords:** neglected tropical diseases, Spain, immunosuppression, migration, neurotoxoplasmosis, neurocysticercosis, central nervous system infections, *Toxoplasma gondii*, *Taenia solium*

## Abstract

Parasitic infections of the central nervous system (CNS) constitute a diverse group of diseases, many of which the World Health Organization classifies as neglected tropical diseases. This study aimed to analyze trends in diagnosis, clinical profile, and epidemiology of these infections through a retrospective review of cases at a secondary hospital in southern Madrid, Spain, from January 1992 to December 2024. Among the 74 patients diagnosed, 58 (78.4%) had neurotoxoplasmosis (NT), 14 (18.9%) neurocysticercosis (NC), and the remaining two cases were cerebral malaria and disseminated amoebiasis with CNS involvement. Diagnosis primarily relied on neuroimaging, which suggested differences in parasite locations within the CNS. All NT patients were HIV-associated immunocompromised, whereas all NC patients were immunocompetent. NT incidence has declined substantially since the late 20th century, following the introduction of highly active antiretroviral therapy, which has also reduced recurrences and mortality. Conversely, NC shifted from a locally endemic disease to an imported one, with all cases diagnosed after 2001 involving patients from Latin America, reflecting both the success of *Taenia solium* control measures in Spain and the impact of migration. These findings underscore the importance of epidemiological surveillance and control strategies tailored to at-risk populations and the demographic shifts associated with migration.

## 1. Introduction

Although less common than viral or bacterial infections, parasitic infections of the central nervous system (CNS) constitute a diverse group of diseases, many of which the World Health Organization (WHO) classifies as neglected tropical diseases (NTDs) [1]. The most common neuroparasitic infections in clinical practice are neurotoxoplasmosis (NT), caused by *Toxoplasma gondii*, and neurocysticercosis (NC), caused by *Taenia solium* [2].

Neurotoxoplasmosis is the most prevalent parasitic neuroinfection worldwide. It is estimated that approximately 25% of the world’s population is infected with *Toxoplasma gondii* [3]. While immunocompetent individuals typically remain asymptomatic or have mild symptoms, neurotoxoplasmosis poses a serious health risk for immunocompromised patients, particularly HIV-infected individuals and transplant recipients [4].

Neurocysticercosis, caused by the larval stage of *T. solium* (cysticercus), is considered the most prevalent helminthic CNS infection [2] and contributes to approximately 30% of epilepsy cases in endemic regions of Latin America, Asia, and sub-Saharan Africa [5].

In Spain, according to the National Hospital Discharge Database, 81 cases of parasitic neuroinfections were recorded in 2024; NT and NC accounted for most diagnoses, representing 34.6% and 56.8% of cases, respectively, while other parasitic neuroinfections represented the remaining 8.6% [6]. Although *T. gondii* infection remains widely distributed in Spain, a recent systematic review and meta-analysis estimated a pooled human seroprevalence of 32.3% between 1993 and 2023 and 38.2% among immunocompromised populations [7]. However, hospitalizations due to NT have declined in recent decades [8]. Conversely, NC, which was once endemic in Spain until the late 20th century, has re-emerged as an imported disease in the early 21st century [9]. Therefore, this study aimed to analyze the trends in diagnosis, clinical manifestations, management, and epidemiology of neuroparasitic infections in a secondary hospital in the south of the Madrid region, Spain.

## 2. Materials and Methods

### 2.1. Study Design and Setting

This was a retrospective study conducted at Severo Ochoa University Hospital (SOUH), a secondary hospital serving as the reference for two municipalities in the south of the Madrid region (Spain): Leganés (195,734 inhabitants) and Fuenlabrada (190,076 inhabitants) until 2004, when Fuenlabrada Hospital opened. After that, SOUH remained the reference hospital only for the municipality of Leganés.

All patients diagnosed with a parasitic CNS infection between January 1992 and December 2024 were included. The case definition was based on clinical signs, exposure factors, and radiologic and/or microbiologic findings. Only the first infection episode per patient was considered.

### 2.2. Diagnostic Procedures

Clinical suspicion was supported by detection of the parasite or by the presence of antibodies against it. Serological testing was used only in patients with NT or NC. Different diagnostic tests were used depending on their availability and the clinician’s criteria. Not all tests were performed on every patient. The diagnosis of cerebral malaria was based on direct parasite detection by thick blood smear, whereas the diagnosis of cerebral amoebiasis was confirmed by microscopy and *Entamoeba histolytica*-specific PCR, performed at a reference laboratory (National Centre for Microbiology, Madrid, Spain).

Neuroimaging, including computed tomography (CT) or magnetic resonance imaging (MRI), was performed in the vast majority of patients. For NT, anti-*T. gondii* antibodies were detected by enzyme-linked immunosorbent assay (ELISA) or indirect chemiluminescent immunoassay (CLIA); the specific commercial platforms varied over the study period due to changes in laboratory technology, and manufacturer information could not be retrieved retrospectively for all cases, except for the adoption of the Alinity CLIA system (Abbott Laboratories, Abbott Park, IL, USA), from 2022 onward. For NC, anti-*T. solium* antibodies were detected by an *in house* EIA at the reference laboratory (National Centre for Microbiology, Majadahonda, Spain). For the remaining cases, diagnosis was supported by additional microbiological methods, including microscopy and molecular testing.

### 2.3. Variables

Data were obtained from clinical records and the Microbiology database for all patients meeting the case definition. Collected data included: date, age, sex, country of birth, length of residence in Spain, diagnostic techniques, parasite location within the CNS, immunocompromising conditions (such as HIV, oncological/hematological illnesses, or immunosuppressive treatments), hospital admission, length of stay, symptoms, coinfections, treatment used, outcome, and laboratory parameters.

### 2.4. Data Analysis and Statistical Methods

Statistical analyses were performed using R: a language and environment for statistical computing (version 4.4.0, R Foundation for Statistical Computing, Vienna, Austria). Categorical variables were analyzed using either a chi-squared test or Fisher’s exact test. Continuous variables were first tested for normality using the Shapiro–Wilk test. For normally distributed variables, homogeneity of variances was assessed using Levene’s test; ANOVA was applied when variances were equal, and the Kruskal–Wallis test was used otherwise. The Kruskal–Wallis test was also applied to non-normally distributed variables. Statistical significance was set at *p* < 0.05. For laboratory parameters that were not available for all patients, those patients were excluded from the analysis of the corresponding variables. When analyzing coinfections, symptoms, or laboratory abnormalities, a single patient may contribute multiple observations to a given variable because multiple conditions may be present simultaneously. For incidence calculation, the following equation was used: incidence per 100,000 inhabitants = (number of cases × 100,000)/total population in the area of study.

### 2.5. Ethics

This study was approved by the Clinical Research Ethics Committee of Severo Ochoa University Hospital (C.E.I.m) on 25 September 2024 under code: A-1624-2024.

## 3. Results

During the study period, 74 patients were diagnosed with a CNS parasitic infection, of whom 58 (78.4%) presented with NT, 14 (18.9%) with NC, one with *Plasmodium falciparum* cerebral malaria, and one with disseminated amoebiasis presenting with brain abscesses, caused by *Entamoeba histolytica*.

Parasitic infections of the CNS were diagnosed predominantly through neuroimaging studies (Table 1). These studies also allowed determination of the parasite location within the CNS (Table 2). When both isolated and multiple-site lesions were considered, basal ganglia involvement was significantly more frequent in NT than in NC patients (18/58, 31.0% vs. 0/14, 0%; *p* = 0.02). Conversely, subarachnoid space involvement was observed in 3/14 NC patients (21.4%) and in none of the NT patients (*p* = 0.006).

Serological testing confirmed the presence of anti-*Toxoplasma* IgG antibodies in 35 of the 42 NT patients tested. Meanwhile, anti-*T. solium* IgG antibodies were detected in 7 of the 9 NC patients tested (Table 1).

No significant association was observed between parasite anatomical location and clinical manifestations among patients with a single CNS location in either the NT or NC group.

Epidemiological characteristics showed that 49 (84.5%) NT patients and 6 (42.9%) NC patients were originally from Spain. All patients diagnosed with NT (*n* = 45) or NC (*n* = 6) before 2002 were Spanish-born (Table S1). From 2002 onwards, among 13 NT patients, 69.2% (*n* = 9) were foreign-born, originating from Equatorial Guinea (n = 4), Nigeria (*n* = 2), Colombia (*n* = 1), Bolivia (*n* = 1), and Romania (n = 1). Likewise, all NC patients diagnosed after 2001 (*n* = 8) were immigrants from Latin America, including Ecuador (*n* = 6), Bolivia (n = 1), and Peru (*n* = 1).

The median age was 33.1 years among NT patients and 41.6 years among NC patients; females accounted for 32.8% and 78.6% of NT and NC patients, respectively (Table 3).

Spanish-born NC patients were significantly older than their Latin American counterparts (median age, 61.5 years [IQR 13.6] vs. 31.4 years [IQR 14.4]; *p* = 0.009). No significant age difference was observed between Spanish- and foreign-born NT patients (median age, 33.1 years [IQR 8.8] vs. 32.8 years [IQR 9.3]; *p* = 0.79).

Immune status differed markedly between the study groups. All NT patients were people living with HIV and were considered immunosuppressed; 49 (84.5%) of them had at least one other coinfection (Table 3). In contrast, all NC patients were immunocompetent, with only one (7.1%) having at least one coinfection.

The most frequent clinical manifestations among NT patients were paresis (43.1%), motor impairment (41.4%), and behavioral impairment (32.8%). Among NC patients, headaches were the most frequent manifestation (64.3%), followed by motor impairment and seizures (35.7% each); headaches were significantly more frequent in NC than in NT patients (*p* = 0.008) (Table 3).

All patients diagnosed with a neuroparasitic infection required hospitalization. The median length of hospital stay was significantly longer for NT patients than for NC patients, 17 days (IQR = 15.5) vs. 9 days (IQR = 8) (*p* = 0.009). Treatment regimens for both NT and NC showed no relevant changes over the study period. A combination of pyrimethamine plus sulfadiazine was used in 48 (82.8%) NT patients, pyrimethamine plus clindamycin in 5 (8.6%), and pyrimethamine plus atovaquone in 1 (1.7%). Anti-*Toxoplasma* treatment was not documented in the medical records of three patients (5.2%), while one patient (1.7%) did not receive treatment because of an imminent fatal outcome.

Albendazole plus dexamethasone was the most frequently prescribed regimen for NC patients, being administered to nine (64.3%). One patient (7.1%) received a combination of albendazole, praziquantel, and dexamethasone. Two patients (14.3%) did not receive antiparasitic treatment because only calcified cysts were present, while treatment information was unavailable for the remaining two patients (14.3%). Five NC patients (35.7%) also required antiepileptic therapy.

Most patients with NT (*n* = 37, 63.8%) or NC (*n* = 11, 78.6%) achieved complete recovery after treatment. Neurological sequelae developed in six NT patients (10.4%) and three NC patients (21.4%). Recurrence occurred in eight NT patients (13.8%), whereas no recurrences were observed in NC patients. Seven NT patients (12.1%) died, while no deaths occurred among NC patients. Notably, all deaths and seven of the eight NT recurrences occurred before 2000. After 2000, only a single NT recurrence was recorded in 2002.

**Figure 1:**
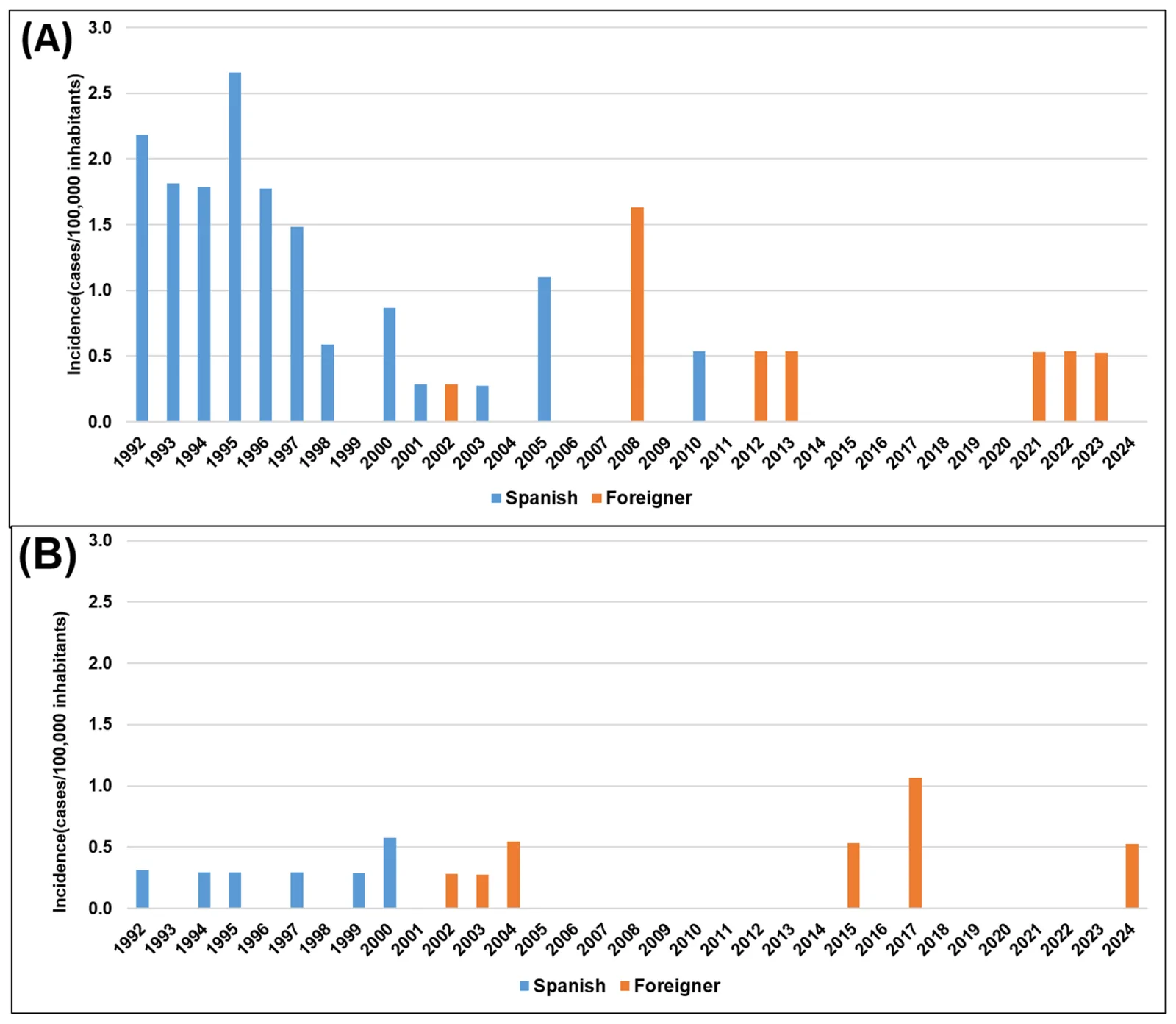
Annual incidence of: (**A**) neurotoxoplasmosis and (**B**) neurocysticercosis in Spanish and foreign-born patients, 1992–2024.

Analysis of temporal trends showed that NT incidence declined steadily from the late 20th century onward (Figure 1A), whereas NC incidence showed a discontinuous pattern, with cases clustered in the early and later parts of the study period (Figure 1B).

Regarding the patient with cerebral malaria, a 3-year-old male was diagnosed in 2003 after traveling to Nigeria. The diagnosis was established based on neurological manifestations in the context of severe malaria, confirmed by thick blood smear examination, which revealed 20% parasitemia. The patient underwent an exchange transfusion in addition to treatment with quinine sulfate and clindamycin, achieving complete recovery after 10 days of hospitalization.

The patient with cerebral amoebiasis was a 39-year-old male who acquired the infection in Spain in 2021. CT scan revealed brain abscesses in the left basal ganglia and right temporal lobe. The diagnosis was confirmed by microscopic examination of abscess material and by *E. histolytica*-specific PCR. The patient was treated with intravenous metronidazole and was discharged after a 16-week stay in the intensive care unit (ICU).

## 4. Discussion

This study presents a comprehensive, long-term, hospital-based review of parasitic CNS infections in southern Madrid, characterizing their diagnostic, clinical, and epidemiological profiles, with NT and NC accounting for the majority of cases.

Although neuroimaging played a central role in diagnosing both NT and NC, MRI—which has historically shown greater sensitivity than CT for detecting NT lesions and viable cysticerci [5,10]—was used less often than CT in both groups. A similar predominance of CT over MRI was reported in 2026 in an NT cohort, with CT alone used in 24/63 patients compared with 9/63 for MRI [11]. Although both imaging techniques were available at SOUH throughout the study period, CT could typically be obtained more rapidly than MRI, particularly in the emergency setting, where prompt diagnosis and management were required. This preference likely reflects clinical decision-making that prioritizes diagnostic speed over sensitivity in acute presentations, rather than a limitation in equipment availability.

Beyond confirming the diagnosis, neuroimaging enabled characterization of the parasite’s location within the CNS (Table 2). Although both NT and NC lesions most frequently involved the brain cortex, overall basal ganglia involvement—considering both isolated and multiple-site lesions—was significantly more frequent in NT than in NC, consistent with earlier neuroimaging studies which identified the basal ganglia as the most common site of toxoplasmic encephalitis in 75% of cases [12]. Conversely, cysticerci were found in the subarachnoid space in 21.4% of NC cases (*p* = 0.006), constituting the so-called subarachnoid NC, a proportion consistent with the 26.1% reported in a Spanish multicenter cohort in 2021 [13].

Parasite anatomical location within the CNS has previously been associated with specific clinical manifestations in both NT and NC. For instance, in NT, basal ganglia involvement has been linked with movement disorders [14]. Similarly, in NC, parenchymal disease typically presents with seizures or headache, whereas ventricular and subarachnoid involvement are associated with hydrocephalus, meningitis, or focal neurological deficits [15]. However, our data showed no significant association between anatomical location and clinical manifestations, which may be attributable to the limited sample size, emphasizing the need for further studies into CNS parasite distribution and its clinical relevance.

Although not sufficient as a stand-alone diagnostic test, serological testing also played a role in diagnosing neuroparasitic infections. In our cohort, 83.3% of NT patients tested positive for anti-*Toxoplasma* IgG antibodies, consistent with the fact that the disease usually represents reactivation of latent infection in immunocompromised hosts. However, seven patients (16.7%) were seronegative, a proportion slightly higher than but close to the 3–15% range reported by Vidal (2019) [16]. This finding supports the recognized limitation that anti-*Toxoplasma* IgG may be absent in some patients with HIV-related cerebral toxoplasmosis, potentially reflecting impaired humoral immunity secondary to HIV infection, although recent exposure before the development of a detectable specific antibody response cannot be ruled out.

Among NC cases, 77.8% of patients were seropositive, with two testing negative despite compatible neuroimaging findings. The sensitivity of *T. solium* serological tests depends on the number, location, and developmental stage of the cysticerci [17,18,19], with reduced sensitivity particularly reported in patients with calcified lesions, where ELISA sensitivity may be as low as 7–13% [20]. One of the seronegative patients presented with a calcified cysticercus, which likely explains the negative result, while the other had two uncalcified intraparenchymal cysticerci, a presentation in which serological sensitivity may also be limited. These findings emphasize that negative serology does not exclude NC when neuroimaging findings are compatible.

A decline in NT incidence was observed from the late 20th century onwards (Figure 1A), consistent with other Spanish studies, reporting a 76.8% reduction in toxoplasmosis-related hospitalizations between 1997 and 2018 [8], and a similar downward trend in *T. gondii* infection among hospitalized people living with HIV, with admissions decreasing by 85.9% between 1997 and 2015 in a Spanish review [21]. This decrease coincided with the introduction of highly active antiretroviral therapy (HAART) for HIV treatment, which improves immune status and reduces the incidence of opportunistic infections, including symptomatic NT, as reported in several early 2000s studies [22,23,24]. These findings support the role of HAART not only in reducing the incidence of opportunistic infections associated with HIV, but also in improving the prognosis of affected patients, as reflected by the fact that all NT-related deaths and the majority of recurrences occurred before 2000, with only a single recurrence recorded thereafter (Table S1). This effect does not appear to be attributable to changes in antiparasitic treatment for NT, which showed no relevant changes over time, with the combination of pyrimethamine and sulfadiazine remaining the most commonly used regimen, in agreement with the recent cohort reported by van Deuzen et al. in the Netherlands [11]. Although HAART was probably the main driver of the observed decline in NT, the decreasing overall seroprevalence of *T. gondii* in Spain—attributed to improved hygienic management of domestic cats, changes in meat consumption habits, and food safety measures—may also have contributed to the lower number of diagnosed NT cases over time [7].

Despite advances in NT control in our setting, the disease remains a major public health concern. A 2015 meta-analysis estimated that *T. gondii* infection accounts for approximately 10–20% of HIV-related deaths worldwide [25], particularly in low-resource settings where access to HAART remains limited [26]. In our cohort, the decline in Spanish-born NT cases after 2002 was accompanied by an increasing proportion of foreign-born patients (Figure 1A), consistent with findings from the Netherlands, where foreign-born individuals accounted for 71% of cerebral toxoplasmosis cases between 2000 and 2024 [11]. This demographic shift likely reflects the combined effect of improved HIV control and declining *Toxoplasma* seroprevalence in Spain, together with migration-related vulnerabilities affecting access to timely HIV diagnosis and treatment.

Importantly, HIV infection among migrants should not be interpreted as exclusively imported. Evidence from Spain and other European countries indicates that a substantial proportion of HIV infections among Latin American migrants may be acquired after migration [27,28]. Therefore, the foreign-born NT cases observed in this cohort should be understood as reflecting both changing migration-related epidemiology and potential post-migration barriers to early HIV diagnosis, linkage to care, and sustained treatment.

The temporal pattern of cysticercosis incidence showed cases in the first part of the study period, followed by a prolonged interval with no diagnoses between 2005 and 2014 (Figure 1B). A decline from approximately 2007 onwards has previously been reported at the national level [29]; however, our data suggest that this decrease may have occurred earlier in our area, consistent with region-specific data for the Community of Madrid reported in the same study [29]. This trend may partly reflect changes in population dynamics, particularly variations in Latin American migration during and after the 2008 economic crisis [30]. In Leganés, the Latin American population remained relatively stable between 2010 and 2015 and increased again from 2015 onwards (Figure S1) [31], coinciding with the reappearance of cysticercosis cases in our cohort (Figure 1B).

The influence of Latin American migration on the epidemiology of cysticercosis in our setting is clearly reflected in changes in the patient profile over time. More than half of the cases occurred in Latin American patients, and the beginning of the 21st century marked a shift from predominantly Spanish-born to Latin American cases (Figure 1B). A similar pattern was previously reported in Madrid, where only 15% of patients diagnosed between 1980 and 1989 were foreign-born, compared with 80% of those diagnosed between 1990 and 2002, nearly all of whom were of Latin American origin [9]. This shift is consistent with the high endemicity of cysticercosis in Latin America [5], together with the progressive interruption of autochthonous transmission in Spain following improvements in sanitation and swine husbandry [32]. In this context, Spanish-born patients diagnosed up to 2000 may represent the final stages of locally acquired disease in our area. Their significantly older age compared with Latin American patients, 61.5 versus 31.4 years, respectively, further supports the transition from a historically endemic disease to a predominantly imported one. Although control of taeniasis and cysticercosis has improved in several Latin American settings, with the regional interruption of *T. solium* transmission in northern Peru providing a well-documented example [33], the impact of these advances on NC incidence may only become apparent after several years, given the potentially long interval between initial infection and clinical manifestation.

We also identified a case of cerebral malaria in a Spanish-born child of Nigerian parentage who had traveled to Nigeria without malaria chemoprophylaxis. Despite presenting with severe malaria, including 20% parasitemia and an estimated mortality of 15–25% even with appropriate treatment [34], the patient made a full recovery. Unlike adults living in endemic areas, who may develop partial immunity against severe malaria through repeated exposure, children born or raised in non-endemic settings do not acquire or maintain such protection and are therefore at increased risk of severe disease when traveling to endemic areas [35]. This case highlights the importance of pre-travel counseling and malaria chemoprophylaxis, particularly for children visiting malaria-endemic regions.

Additionally, we reported a case of disseminated amoebiasis with CNS involvement, presenting as brain abscesses in an immunocompetent Spanish man with no history of international travel [36]. Although amoebiasis is more prevalent in low-income countries, it has a worldwide distribution [37] and should therefore be considered in the differential diagnosis of compatible clinical presentations, even in patients without travel to endemic areas.

This study had certain limitations. First, its retrospective design meant that diagnostic testing was not performed uniformly across all patients, but rather according to clinical judgment and test availability, potentially introducing heterogeneity in case assessment and limiting comparability between patients. Nevertheless, all diagnostic methods used had been previously validated and are widely accepted in clinical practice. Second, the specific commercial kits and platforms used for serological testing could not be identified retrospectively for most of the study period, as laboratory records only allowed confirmation of the assay type (ELISA or CLIA), not the manufacturer; this may have introduced additional variability in antibody detection that could not be fully accounted for. Third, some clinical and laboratory data were missing because of the long study period and reliance on historical medical records.

Overall, this study provides a long-term overview of the changing epidemiology of parasitic CNS infections in southern Madrid. Over more than three decades, NT and NC showed marked changes in incidence and patient profile, shifting from conditions that mainly affected Spanish-born individuals to infections increasingly diagnosed among foreign-born populations. These findings illustrate how advances in disease control, public health measures, HIV management, and migration have reshaped the epidemiology of neuroparasitic diseases. In addition, maintaining clinical awareness of these infections remains essential, particularly in diverse populations and among patients with relevant epidemiological or immunological risk factors.

## Figures and Tables

**Table 1:** Techniques employed for the diagnosis of NT and NC.

Technique	NT (*n* = 58)	NC (*n* = 14)	*p*
Imaging technique			
CT scan, *n* (%)	46 (79.3)	6 (42.9)	0.01
MRI, *n* (%)	5 (8.6)	4 (28.6)	0.07
CT scan + MRI, *n* (%)	6 (10.3)	4 (28.6)	0.09
Unknown, *n* (%)	1 (1.7)	0 (0)	1
Laboratory technique			
anti-*Toxoplasma* IgG, *n*/*n* (%)	35/42 (83.3)	-	-
anti-*T. solium* IgG, *n*/*n* (%)	-	7/9 (77.8)	-

NT, neurotoxoplasmosis; NC, neurocysticercosis; CT scan, computed tomography scan; MRI, magnetic resonance imaging.

**Table 2:** Distribution of CNS parasite locations in NT and NC cases.

Parasite Location	NT (*n* = 58)	NC (*n* = 14)	*p*
Overall involvement			
Any basal ganglia involvement ^1^	18 (31.0)	0 (0)	0.02
Any subarachnoid space involvement ^1^	0 (0)	3 (21.4)	0.006
Single anatomical location			
Brain cortex, *n* (%)	22 (37.9)	6 (42.9)	0.8
Basal ganglia, *n* (%)	10 (17.2)	0 (0)	0.2
Cerebellum, *n* (%)	2 (3.4)	0 (0)	1
Subarachnoid space, *n* (%)	0 (0)	2 (14.3)	0.04
Brainstem, *n* (%)	1 (1.7)	0 (0)	1
Thalamus, *n* (%)	1 (1.7)	0 (0)	1
Multiple anatomical locations, *n* (%)	16 (27.6)	3 (21.4)	0.7
Brain cortex + Basal ganglia, *n* (%)	7 (43.8)	0 (0)	-
Brain cortex + Cerebellum, *n* (%)	3 (18.8)	1 (33.3)	-
Brain cortex + Thalamus, *n* (%)	2 (12.5)	0 (0)	-
Brain cortex + Subarachnoid space, *n* (%)	0 (0)	1 (33.3)	-
Basal ganglia + Thalamus, *n* (%)	1 (6.3)	0 (0)	-
Multiple (≥3) locations, *n* (%)	3 (18.8)	1 (33.3)	-
Unknown, *n* (%)	6 (10.3)	3 (21.4)	0.4

NT, neurotoxoplasmosis; NC, neurocysticercosis. ^1^ Overall involvement includes both isolated involvement and involvement as part of multiple anatomical locations.

**Table 3:** Clinical and epidemiological characteristics of the NT and NC patients.

	NT (*n* = 58)	NC (*n* = 14)	*p*
Age			0.2
Median age (IQR)	33.1 (8.4)	41.6 (27.6)	
Range	17–73	22–77	
Gender			0.003
Male, *n* (%)	39 (67.2)	3 (21.4)	
Female, *n* (%)	19 (32.8)	11 (78.6)	
Clinical manifestations			
Motor impairment, *n* (%)	24 (41.4)	5 (35.7)	0.8
Paresis, *n* (%)	25 (43.1)	4 (28.6)	0.4
Cephalalgia, *n* (%)	14 (24.1)	9 (64.3)	0.008
Perception/Expression impairment, *n* (%)	7 (12.1)	3 (21.4)	0.4
Behavioural impairment, *n* (%)	19 (32.8)	3 (21.4)	0.5
Seizures, *n* (%)	12 (20.7)	5 (35.7)	0.3
General symptoms, *n* (%)	16 (27.6)	1 (7.1)	0.2
Coinfection			
Bacteria ^1^, *n* (%)	20 (34.5)	0 (0)	0.008
HIV, *n* (%)	58 (100)	0 (0)	<0.001
Virus (non-HIV) ^2^, *n* (%)	33 (56.9)	0 (0)	<0.001
Fungi ^3^, *n* (%)	18 (31)	0 (0)	0.01
Parasites ^4^, *n* (%)	2 (3.5)	1 (7.1)	0.5
Outcome			
Recovery, *n* (%)	37 (63.8)	11 (78.6)	0.4
Recurrence, *n* (%)	8 (13.8)	0 (0)	0.3
Sequelae, *n* (%)	6 (10.4)	3 (21.4)	0.3
Death, *n* (%)	7 (12.1)	0 (0)	0.3

^1^ *Mycobacterium tuberculosis*, *n* = 12; *Mycobacterium avium*, *n* = 2; syphilis, *n* = 2; *Campylobacter jejuni* gastroenteritis, *n* = 2; pneumococcal pneumonia, *n* = 1; bacteremia, *n* = 5. ^2^ *Hepatitis C virus*, *n* = 17; *Hepatitis B virus*, *n* = 9; *Cytomegalovirus*, *n* = 7; *Herpes Simplex virus* 1, *n* = 6; *Varicella-Zoster virus*, *n* = 7; *Human Herpesvirus* 8, *n* = 1; *Hepatitis E virus*, *n* = 1. ^3^ Oral candidiasis, *n* = 12; *Pneumocystis jirovecii* pneumonia, *n* = 3; genital candidiasis, *n* = 1; pulmonary aspergillosis, *n* = 1; cutaneous candidiasis, *n* = 1; pityriasis versicolor, *n* = 1. ^4^ NT: cryptosporidiosis, *n* = 1; visceral leishmaniasis, *n* = 1. NC: ocular toxoplasmosis, *n* = 1.

## Data Availability

The data that support the findings of this study are not publicly available due to privacy and data protection restrictions. However, the data are held by the corresponding author and may be shared upon reasonable request, subject to appropriate ethical and legal considerations.

## Supplementary Materials

The following supporting information can be downloaded at: https://www.mdpi.com/article/10.3390/pathogens15101027/s1, Table S1: Clinical and epidemiological characteristics of patients with neurotoxoplasmosis according to two periods: 1992–2000 and 2001–2024; Figure S1: Population trends in Leganés between 1996 and 2022. (A) Total population according to Spanish or foreign country of birth. (B) Foreign-born population according to continent of birth.

## Abbreviations

The following abbreviations are used in this manuscript:

CNS	Central nervous system
WHO	World Health Organization
NT	Neurotoxoplasmosis
NC	Neurocysticercosis
