# Supplementary material for: Evolution of Neuroparasitic Infections in the South of Madrid Region, Spain: A Hospital-Based Study
Source: Pathogens. 2026 Sep 30;15(10):1027. doi: 10.3390/pathogens15101027 (PMC13708643; doi:10.3390/pathogens15101027)
Supplement: Supplementary file 1 [file pathogens-15-01027-s001.zip › pathogens-4568016-supplementary.pdf]

Table S1. Clinical and epidemiological characteristics of patients with neurotoxoplasmosis according to two periods: 1992–2000 and 2001–2024

Variable	1992–2000 (n=44)	2001–2024 (n=14)	p value
**Sex**			0.19
Male, n (%)	32 (72.7%)	7 (50.0%)	
Female, n (%)	12 (27.3%)	7 (50.0%)	
**Origin**			<0.001
Spanish, n (%)	44 (100.0%)	5 (35.7%)	
Foreign-born, n (%)	0 (0.0%)	9 (64.3%)	
**Age**			0.11
Age, median (IQR)	32.8 (7.9)	36.7 (12.1)	
**IgG serology**			0.65
Positive, n/n (%)	24/30 (80.0%)	11/12 (91.7%)	
Negative, n/n (%)	6/30 (20.0%)	1/12 (8.3%)	
**Imaging test**			<0.001
CT scan only, n (%)	40 (90.9%)	6 (42.9%)	
MRI, n (%)	2 (4.6%)	3 (21.4%)	
MRI + CT scan, n (%)	1 (2.3%)	5 (35.7%)	
Unknown, n (%)	1 (2.3%)	0 (0.0%)	
**Outcome**			0.03
Cure, n (%)	28 (63.6%)	9 (64.3%)	
Death, n (%)	7 (15.9%)	0 (0.0%)	
Relapses, n (%)	7 (15.9%)	1 (7.1%)	
Sequelae, n (%)	2 (4.5%)	4 (28.6%)	
**Treatment**			0.42
Pyrimethamine/sulfadiazine, n (%)	35 (79.5%)	13 (92.9%)	
Other, n (%)	9 (20.5%)	1 (7.1%)	

Percentages were calculated among patients with available serological results. Serology was unavailable or not performed in 14 patients diagnosed during 1992–2000 and in 2 patients diagnosed during 2001–2024.

A)

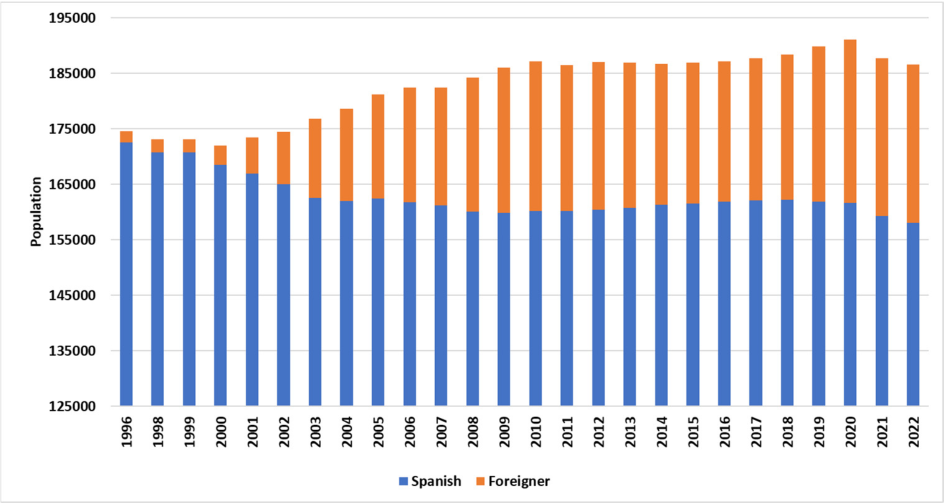

B)

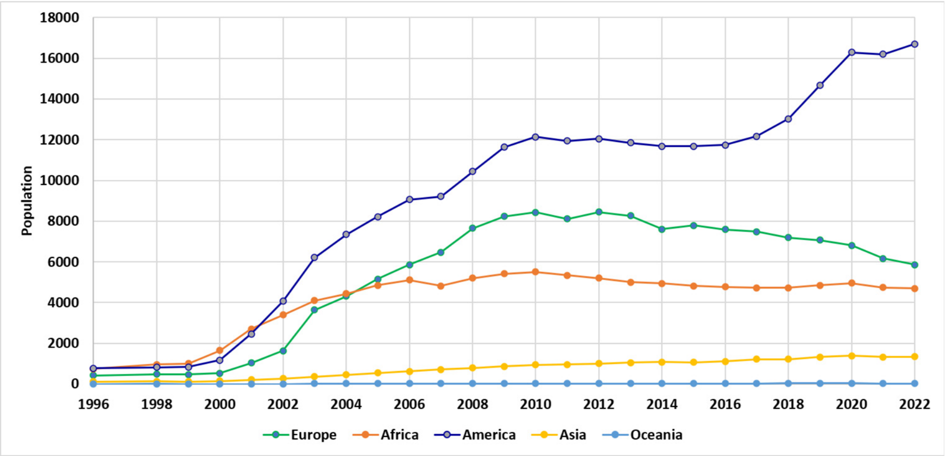

Figure S1. Population trends in Leganés between 1996 and 2022. (A) Total population according to Spanish or foreign country of birth. (B) Foreign-born population according to continent of birth.
